# Echinococcus P29 Antigen: Molecular Characterization and Implication on Post-Surgery Follow-Up of CE Patients Infected with Different Species of the *Echinococcus granulosus* Complex

**DOI:** 10.1371/journal.pone.0098357

**Published:** 2014-05-22

**Authors:** Ghalia Boubaker, Bruno Gottstein, Andrew Hemphill, Hamouda Babba, Markus Spiliotis

**Affiliations:** 1 Institute of Parasitology, University of Berne, Berne, Switzerland; 2 Graduate School for Cellular and Biomedical Sciences, University of Berne, Berne, Switzerland; 3 Department of Clinical Biology B, Laboratory of Parasitology and Mycology, University of Monastir, Monastir, Tunisia; Aga Khan University Hospital Nairobi, Kenya

## Abstract

The protein P29 is a potential serological marker for post-treatment monitoring of cystic echinococcosis (CE) especially in young patients. We now have demonstrated that P29 is encoded in the *Echinococcus* genus by a single gene consisting of 7 exons spanning 1.2 kb of DNA. Variability of the *p29* gene at inter- and intra-species level was assessed with 50 cDNA and 280 genomic DNA clones isolated from different *E. granulosus* s.l. isolates (*E. granulosus* sensu stricto (G1), *E. equinus* (G4), *E. ortleppi* (G5), *E. canadensis* (G6), *E. canadensis* (G7) and *E. canadensis* (G10)) as well as four *E. multilocularis* isolates. Scarce interspecies polymorphism at the *p29* locus was observed and affected predominantly *E. granulosus* s.s. (G1), where we identified two alleles (A1 and A2) coding for identical P29 proteins and yielding in three genotypes (A1/A1, A2/A2 and A1/A2). Genotypic frequencies expected under Hardy-Weinberg equilibrium revealed a high rate of heterozygosity (47%) that strongly supports the hypothesis that *E. granulosus* s.s. (G1) is predominantly outbreeding. Comparative sequence analyses of the complete *p29* gene showed that phylogenetic relationships within the genus *Echinococcus* were in agreement with those of previous nuclear gene studies. At the protein level, the deduced P29 amino acid (AA) sequences exhibited a high level of conservation, ranging from 97.9% AA sequence identity among the whole *E. granulosus* s.l. group to 99.58% identity among *E. multilocularis* isolates. We showed that P29 proteins of these two species differ by three AA substitutions without implication for antigenicity. In Western-blot analyses, serum antibodies from a human CE patient infected with *E. canadensis* (G6) strongly reacted with recombinant P29 from *E. granulosus* s.s. (G1) (recEg(G1)P29). In the same line, human anti-Eg(G1)P29 antibodies bound to recEcnd(G6)P29. Thus, minor AA sequence variations appear not to impair the prognostic serological use of P29.

## Introduction

Human echinococcosis is a severe disease caused by infection with the metacestode stage of tape-worms belonging to the genus *Echinococcus* (family *Taeniidae*) [1]. *E. granulosus* sensu lato (*E. granulosus* s.l.) and *E. multilocularis* are the most common species infecting humans, hereby causing cystic echinococcosis (CE) or alveolar echinococcosis (AE), respectively.

According to the guidelines of the WHO informal echinococosis working group [2], recommended treatment options for human echinococcosis include (i) full surgical resection of the parasitic lesions followed by chemotherapy for operable cases and (ii) long-term chemotherapy as the only treatment option for inoperable cases or cases where only partial resection was possible. In the post-surgical or medical echinococcosis therapy program, the risk of recurrence represents the major problem [3]–[5]. In this respect, post-treatment follow- up of patients for several years is mandatory to detect potential recurrences as soon as possible. Standardized and structured follow-up methodology (serological and imaging procedures) is essential for clinicians to carry out a reliable prognosis for the patients. Especially for CE, an agreement on which tests should be used, and how these tests should be applied and interpreted, is still lacking.

The P29 protein was first described by González et al. [6] as a novel 29 kDa antigen from *E. granulosus* protoscoleces, while looking for parasite antigens distinct from those present in hydatid cyst fluid. Subsequently, the same protein (EgP29) has been characterized within *E. granulosus* s.s. (G1) protoscolex-derived soluble somatic antigen, as a biomarker for monitoring of CE patients [7]. A recombinant version of P29 (recEg(G1)P29) has been generated and appeared to be a prognostically useful tool for post-surgical monitoring of especially young CE patients, as P29-specific antibodies assessed against native and recEg(G1)P29 antigen gradually disappeared within approximately six months after surgery in a high proportion of cured CE patients [8]. This rapid decrease of antibody concentrations was not observed for antibodies raised against other *Echinococcus* antigens [9], maybe with the exception of B2t [10], a peptide derived from AgB2, that showed also a relatively good association between antibody kinetics and cure of CE. In conclusion, the determination of anti-P29 antibody levels in the post-surgical follow-up of CE patients could therefore represent a valuable prognostic tool for the clinical management of CE cases. Standardization of a serological test based on recEg(G1)P29 is of high interest for post treatment follow-up, especially of young CE patients [8]. However, the question that has not been addressed so far is whether the designed recombinant form originating from *E. granulosus* s.s. (G1) will also be recognized by human sera of patients that are infected with a different species and/or genotype of the *E. granulosus* s.l. complex. All recombinant proteins/antigens used for immunodiagnosis or vaccine development need to be investigated for their degree of genetic conservation (or diversification). Antigenic variation based on allelic polymorphisms and/or multigene family encoding proteins can influence the prognostic and protective potential of any recombinant antigen. Currently, ten distinct genotypes (G1-G10) of *E. granulosus* s.l. have been documented and were named according to their most commonly identified intermediate host. The majority of them were known to infect humans, with exception of the G4 horse strain which has not been reported to be zoonotic in the literature. Further phylogenetic analyses revealed that *E. granulosus* s.l. was not a uniform species and has now been split into *E. granulosus* sensu stricto (s.s.) (G1/G2/G3), *E. equinus* (G4), *E. ortleppi* (G5), and *E. canadensis* (G6–G10) [11].

In *E. granulosus* s.l. several genes that encode diagnostic antigens have been shown to form part of multigene families. As an example, recent studies have demonstrated that antigen B (AgB), which has a high diagnostic value for CE, is encoded by a highly polymorphic multigene family consisting of at least five (AgB1–AgB5) variably expressed major gene clusters [12]–[18]. The differences between putative isoforms encoded by the five EgAgB groups can reach 81% at the amino acid (AA) level [19], [20], and comparative studies of antigen B subunits (EgAgB1 and EgAgB2) derived proteins/peptides revealed remarkable differences in the diagnostic performances [21]. Thus, on one hand, this large antigenic variation makes it difficult to standardize a recombinant antigen B based assay. On the other hand, diversity of this protein may also limit effectiveness of any application mode as a vaccine candidate. This potential problem of antigenic variability was properly assessed for EG95-1G1-vaccine, a promising *E. granulosus* vaccine candidate [22], [23]. Recombinant EG95-1G1-based vaccination has yielded a very high protection in ruminants against *E. granulosus* genotype G1 infection. The corresponding gene, *Eg EG95-1*, belongs to a small multigene family including five members [24]. Variation neutrality was challenged for EG95 genes [22] and theoretically calculated data indicated a genetic polymorphism of the EG95 antigen-coding genes among different genetically characterized strains of *E. granulosus* [23]. Rojas et al. [25], [26] showed that this variability affects the antigenicity of EG95 antigen, by demonstrating that recombinant protein EG95-1G6 (*E. canadensis* (G6)) was not recognized by antibodies raised by sheep vaccinated with EG95-1G1. Genetic polymorphism in diagnostic/vaccine candidates was also reported for other parasites including protozoa (e.g. *Trypanosoma cruzi* [27]), trematodes (*Schistosoma japonicum* [28]), cestodes (e.g. *Taenia saginata* [29]) or nematodes (e.g., *Trichostrongylus colubriformis* [30]).

In the present study, we experimentally addressed the genetic polymorphism of the *p29* gene within a relatively large selection of members of the *Echinococcus* genus, in order to predict the reliability of this serological tool upon use for CE in humans infected with different genotypes of *E. granulosus* sensu lato.

## Materials and Methods

### Ethical statement

All parasite samples of animal origin were obtained from existing collections. Respective samples were collected in the frame of governmentally regulated meat inspection at public abattoirs, and specimen were sent in to the laboratory of parasitology for respective parasitological and molecular identification. Parasite samples from human origin were derived from an existing collection of *Echinococcus* DNA biobank at EPS Fattouma Bourguiba hospital in Monastir/Tunisia, designed and approved to be used for basic research studies. The parasite samples were fully anonymized respective to the patients where it came from, thus requiring no further approval. All parasite samples (animal and human origin) had already been used in a respectively approved previous study [32].

All clinical human serum samples were collected as part of public health and clinical diagnostic activities, were pre-existing relative to the start of the study, and were examined as anonymous samples. Furthermore, all patients – in the frame of their follow-up assessment, have complied, upon request of their treating clinician, on the use of their serum for experimental serodiagnostic investigations. Baseline data for all samples include only the geographical origin of patients, thus no reference to personal data was recorded. For data evaluation, all samples were anonymized. All serum samples had already been used in two respectively approved previous studies [7], [8]. Ethical review of the study protocol was performed and subsequently approved by the IPA Review Board of the Vetsuisse Faculty of Bern/Switzerland.

### Parasite material, DNA/RNA sample preparation and genotyping

Genomic DNA (gDNA) from *E. equinus* (G4) (Spain), *E. ortleppi* (G5) (Brasil), *E. canadensis* (G6) (Algeria), *E. canadensis* (G7) (Argentina) and *E. canadensis* (G10) (Estonia) as well as (ii) genomic DNA from four *E. multilocularis* isolates originating from different geographical areas (Switzerland, Germany, St. Lawrence Island and Canada) were obtained from the institutional DNA-collection in Bern/Switzerland (See table 1).

**Table 1:** Molecular characterization of *p29* genomic sequence within *Echinococcus* genus.

Sample no.	*Echinococcus* species	Sample origin	Total no of sequenced clones	No. of clones	Sequence type
1	*E. granulosus* s.s. (G1)	Sheep	20	12	*E.g* s.s.(G1) P29A1
				8	*E.g* s.s.(G1) P29A2
2	*E. granulosus* s.s. (G1)	Sheep	20	9	*E.g* s.s.(G1) P29A1
				11	*E.g* s.s.(G1) P29A2
3	*E. granulosus* s.s. (G1)	Sheep	20	18*	*E.g* s.s.(G1) P29A2
4	*E. granulosus* s.s. (G1)	Sheep	20	20	*E.g* s.s.(G1) P29A1
5	*E. granulosus* s.s. (G1)	Sheep	20	8	*E.g* s.s.(G1) P29A1
				10*	*E.g* s.s.(G1) P29A2
6	*E. equinus* (G4)	Horse	20	20	*E.eq* (G4) P29
7	*E. ortleppi* (G5)	Cow	20	19*	*E.ort* (G5) P29
8	*E. canadensis* (G6)	Camel	20	20	*E.can* (G6) P29
9	*E. canadensis* (G7)	Pig	20	20	*E.can* (G7) P29
10	*E. canadensis* (G10)	Deer	20	17	*E.can* (G10) P29
11	*E.multilocularis* (Switzerland)	Human	20	20	*E.m* P29
12	*E.multilocularis* (Germany)	Human	20	20	*E.m* P29
13	*E.multilocularis* (St. Lawrence Island)	Human	20	20	*E.m* P29
14	*E.multilocularis* (Canada)	Human	20	20	*E.m* P29

*In total five clones were rejected because of the bad quality of their sequences (2 clones from sample 3, 2 from sample 5 and 1 clone from sample 7).

These samples were used to sequence the genomic locus of *p29* and to identify species- specific differences. Five samples of protoscoleces derived from different sheep hydatid cysts were collected in a slaughterhouse in Sousse/Tunisia (sheep origin: rural regions close to Sousse) and 34 human samples were harvested from Tunisian patients after surgery (Monastir/Tunisia). From every cyst of animal or human origin, sedimented protoscoleces from one single cyst were fixed in 95% (v/v) ethanol and defined as sample. Genomic DNA was extracted using a standard phenol-chloroform protocol [31], using RNAse A, Proteinase K and a subsequent isopropanol precipitation followed by multiple washes in 75% EtOH prior to drying and dissolving in ddH2O. For all described samples the genotype was determined as *E. granulosus* s.s. (G1) using a recently published multiplex PCR method [32]. Total RNA was extracted from protoscoleces of 5 *E. granulosus* s.s. (G1) and 1 *E. canadensis* (G6) samples respectively isolated from 5 sheep and 1 camel hydatid cysts using the phenol based Peq- Gold RNA Pure kit (peqlab), treated with DNAseI to remove traces of contaminating DNA and finally transcribed into cDNA using M-MLV Reverse Transcriptase (New England Biolabs) and oligo (dT) primers according to the manufacturer's instructions. The 5 sheep samples were used to sequence the cDNA and the gDNA in an *E. granulosus* s.s. (G1) background, and the 34 Tunisian human samples were used to identify allelic differences via multiplex PCR (MAS-PCR, see below).

### *p29* cDNA/gDNA PCR-amplification, cloning and sequencing

To amplify the *p29* genomic DNA sequence from different *Echinococcus* species, the *p29*-cDNA sequence (GenBank, accession no. AF078931) was blasted against the *E. granulosus* genome assembly data base at the Sanger Institute BLAST server (http://www.sanger.ac.uk/cgi-bin/blast/submitblast/Echinococcus). Using the identified genomic *p29* sequence found on the pathogen_EgG_scaffold_0007, we designed the forward primer (Echi gP29F Forward) located at position −47 upstream of the start-ATG and the reverse Primer (Echi gP29R Reverse) located +55 downstream of the stop-TAG. The additional primers cP29-forward starting from the start-ATG of p29 and cP29-reverse located at the TAG-stop codon of *p29*, were used to amplify the protein coding cDNA sequence (732 bp). All used primers are specified in table 2 and all accession numbers are listed at the end of the material and methods part. PCRs were performed in a reaction volume of 20 µl containing 25 ng DNA sample, 0.5 µM primers each (Sigma-Aldrich), 200 µM dNTPs (Promega) and 0.4 units Phusion High-Fidelity DNA polymerase in 1× Phusion HF PCR Buffer (both from New England Biolabs). The cycling conditions were as follows: an initial denaturation step at 98°C for 2 min, 25 cycles (98°C–30 s, 58°C–30 s, 72°C–1.5 min) and a final extension step lasting 5 min at 72°C. The amplified DNA fragments were separated by electrophoresis in a 1% agarose gel and visualized by ethidium bromide staining and subsequent UV excitation. The specific bands were cut from the gel, purified with the high pure PCR purification kit (Roche) and A-overhangs were added with Taq polymerase prior TA-cloning into the pGEM-Teasy vector (Promega). Colonies were screened by colony PCR (Primers: T7-forward, sp6-reverse) and *p29*-positives were cultured overnight in ampicillin containing LB medium prior to plasmid purification using the plasmid miniprep Kit (Qiagen). Plasmids were sequenced from the T7- and SP6 primer sites using the big dye terminator kit on an ABI Prism 3730XL sequencer (Applied Biosystems). Plasmids containing genomic sequences were additionally sequenced with a third primer (Echi P29E5R) designed in the *p29*-exon 5 to cover the internal region (see table2).

**Table 2:** Information of used primers.

Primer name	Primer sequences	Primer position (on genomic sequence of *E. granulosus* (G1) allele 1 (KF528663)	Product size (bp)
1. Primers to amplify *p29* gDNA			
Echi gP29F Forward primer (5′-3′)	TAAGCTGTGGGAACTAGTT	P −47 upstream of the start-ATG	1302
Echi gP29R Reverse primer (5′-3′)	TATGTGAACAAGCTAACAGG	P+55 downstream of the stop-TAG	
2. Primers to amplify *p29* cDNA			
Echi cP29F Forward primer (5′-3′)	ATGTCCGGATTTGACGTTACTAAG	Exon 1: P1–P24	732
Echi cP29R Reverse primer (5′-3′)	CTACTCGCCCAGCATCATCATACT	Exon 7: P1201–P1181	
3. Primers used for sequencing			
Echi P29E5R Reverse primer (5′-3′)	CTGTTCCGCAGTCTTAG	Exon 5: P864–P848	
T7 Forward	TAATACGACTCACTATAGGG		
SP6 Reverse	ATTTAGGTGACACTATAG		
4. MAS-PCR primers			
EgG1P29A1F Forward primer (5′-3′)	CAGCAAGATCATCAC**C** *	Exon2: P179–P194	195
EgG1P29A1R Reverse primer (5′-3′)	AA*G*ACAACACCATATCTTA**G** *	Intron 2: P374–P355	
EgG1P29A2F Forward primer (5′-3′)	ACCCTATTCATTTTTGC**T** *	Intron 3: P525–P542	258
EgG1P29A2R Reverse primer (5′-3′)	TTTCTAAGTGGGATAAAG**G** *	Intron 4: P783–P765	
5. Primers to amplify *p29* cDNA (for protein expression)			
CACC-p29-Forward (5′-3′)	CACCATGTCCGGATTTGACGTTACTAAG		
p29-Reverse (5′-3′)	CTACTCGCCCAGCATCATCATACT		

*Allele specific 3′-base in each primer.

Trans-splicing is a special mechanism of RNA processing in eukaryotes where exons from two different primary RNA transcripts are fused to form mature mRNA molecules. Trans- splicing was described in *Echinococcus* and a spliced leader (SL) that is trans-spliced to echinococcal pre-mRNAs was reported [33]. To determine whether or not the *p29* transcript is transpliced or not with this SL, we carried out a PCR using forward SL-5PR (5′- CACCGTTAATCGGTCCTTAC-3′) [33] and reverse Echi-P29R primer (5′- CTACTCGCCCAGCATCATCATACT-3′) or reverse Echi-P29E5R (5′- CTGTTCCGCAGTCTTAG-3′). PCR was performed as described above (see section *p29* cDNA/gDNA PCR amplification) in a reaction volume of 20 µl containing 2 µl (250 ng) template cDNA. As positive control, we amplified cDNA of the *elp* gene encoding a member of the ezrin/radixin/moesin protein family, that was reported to be trans-spliced [33]. An additional blast search using the *p29* cDNA sequence (GenBank, accession no. AF078931) on an *E. granulosus* spliced-leader cDNA library from the protoscolex stage (http://www.sanger.ac.uk/cgi-bin/blast/submitblast/Echinococcus) was also performed.

### Multiplex allele-specific PCR (MAS-PCR) for analysis of the *p29* alleles within Tunisian *E. granulosus* s.s. (G1) samples

Since sequencing results showed that the *E. granulosus* s.s. (G1) *p29* is present in two allelic forms in Tunisia (A1 and A2), we aimed to determine the genotype frequency and to investigate its possible association with CE infection in humans. Thus, we genotyped a cohort of 34 *E. granulosus* s.s. (G1) isolates obtained from Tunisian human patients using a multiplex allele-specific PCR (MAS-PCR). Primers were designed containing allele-specific 3′-ends to differentiate single nucleotide changes at the specific loci. For human homozygote *E. granulosus* s.s. (G1) isolates (A1/A1 or A2/A2), only one single allele-specific fragment was amplified, yielding a 195 or a 258 bp band, respectively. In case of heterozygote samples (A1/A2), both fragments were amplified (See table 2). All primers used are specified in Table 2, and all accession numbers are listed in an extra chapter at the end of the material and methods part.

MAS-PCRs were performed in a reaction volume of 20 µl containing 20 ng DNA sample, 10 pmol primers each of the allele A1 specific primers EgG1P29A1F and EgG1P29A1R as well as of the allele A2 specific primers EgG1P29A2F and EgG1P29A2R, 100 µM dNTPs and 0.05 units µl-1 GoTaq DNA polymerase in 1× PCR Buffer. The cycling conditions were as follows: an initial denaturation step at 94°C for 2 min, 25 cycles (94°C–30 s, 58°C–30 s, 72°C–30 s) and a final extension step lasting 5 min at 72°C. The amplified DNA fragments were separated by electrophoresis in a 2% agarose gel and visualized by ethidium bromide staining and subsequent UV excitation. Allele frequencies, observed heterozygosity (HO), and heterozygosity expected from Hardy-Weinberg (HW) assumptions (HE) for the *p29* locus were calculated.

### Recombinant expression of P29 protein

Among the members of the *E. granulosus* complex, both *E. granulosus* s.s. (G1) and *E. canadensis* (G6) are the main species involved in human infections worldwide. In this part we tested whether the three AA differences revealed in the P29 protein sequence between these two species might have any serological implications on the use of recombinantly expressed recEg(G1)P29 for the serodiagnosis of CE. The coding sequences for *E. granulosus* s.s. (G1) P29 (Eg(G1)P29) and *E. canadensis (G6)* P29 (Ecnd(G6)P29) were amplified with the primers CACC-*p29*-Forward and p29-Reverse described in table 2. The gel-eluted fragments were cloned into the vector pET-151 (Invitrogen) according to the manufacturer's instructions and sequenced as described above with the primers T7 and Echi P29R Reverse primer. Positive plasmids were transfected into *E. coli* BL21(DE3) and 10 ml of an ON-culture were diluted in 1 liter LB medium containing 100 µg/ml carbencillin. At an OD 600 of 0.5, the expression of recombinant P29 proteins from *E.granulosus* s.s. (G1) (recEg(G1)P29) and *E. canadensis (G6)* (recEcnd(G6)P29) was induced by addition of 1 mM IPTG. After 3 h of induction, the culture was centrifuged (4,000×g, 20 min) and the recombinant expressed proteins were purified under native conditions via their HIS-tags using a Ni-IDA resin (Protino Ni-IDA; Machery-Nagel according to the manufacturer's instructions). Purified recEg(G1)P29 and recEcnd(G6)P29 were checked on a 12% silver staining-stained SDS-PAGE gel and subsequently stored at -20°C until use for serological tests.

### Immunoblotting and competition experiments

RecEg(G1)P29 or recEcnd(G6)P29 proteins were solubilized with SDS loading buffer (1 mol/l Tris HCl, pH 6.8, 2% SDS, 5% 2-β-mercaptoethanol, 10% glycerol) and incubated for 5 min at 95°C. 1 µg RecEgP29 proteins were separated on a 12% SDS-PAGE mini-gel using a preparative comb (length 7 cm) and transferred to a nitrocellulose membrane. Blots were blocked with 1×PBS containing 5% skimmed milk powder and 0.3% Tween 20 overnight at 4°C. The filter was cut into strips (3 mm width), and each strip was individually incubated with one of the 34 *E. granulosus* s.s. (G1) sera, and one strip was incubated with a serum obtained from an *E. canadensis* (G6) CE case (G6 sera are very difficult to get). The sera were diluted 1/100 in PBS (5% skimmed milk powder, 0.3% Tween 20). Strips were incubated overnight at 4°C, then washed 3 times for 5 min with 1×PBS, 0.1% Tween 20 and finally incubated for 2 h at room temperature with an alkaline-phosphatase conjugated goat anti-human IgG antibody (Sigma-Aldrich) diluted 1∶1000 in 1×PBS containing 0.1%Tween 20. Strips were developed in 1 ml BCIP/NBT alkaline phosphatase substrate.

For competition experiments, 1 human *E. granulosus s.s.* (G1) and 1 human *E. canadensis* (G6) serum were diluted 1∶100 in 1×PBS (5% skimmed milk powder, 0.3% Tween 20) and were individually pre-incubated for 30 min at 37°C with recEcnd(G6)P29 (1 µg rec-protein ml^−1^) prior to incubation with the Western-blot strips. Recombinantly expressed Eg14.3.3, prepared earlier [34] was used as a negative control in the same concentration. Western-blots and respective immunological analysis were performed as described above.

### Alignments, phylogenetic tree reconstruction for *p29* and prediction of *p29*-related gene(s) in *Echinococcus* genomes

To detect differences in the cDNA or gDNA sequence of *p29*, the *p29* sequences of *E. granulosus* s.s. (G1), *E. equinus* (G4), *E. ortleppi* (G5), *E. canadensis* (G6), *E. canadensis* (G7), *E. canadensis* (G10) and *E. multilocularis* were aligned using the ClustalW application of BioEdit version 7.0.9.0. All accession numbers are listed in an extra chapter at the end of the material and methods part. Multiple alignments of genomic *p29* sequences were further used for the phylogenetic tree reconstruction. The conversion of the alignment format to PHYLIP was also done with BioEdit. The maximum likelihood was constructed with molecular clock (version 3.69) phylogenetic tree (http://evolution.genetics.washington.edu/phylip/doc/dnamlk.html) using the T-REX web server for inferring, validating and visualizing phylogenetic trees and networks [35]. To address the question if P29 is encoded by a single gene, gene prediction was performed using the sequenced genomes of the *Echinococcus* species *E. granulosus* s.s. (G1) and *E. multilocularis*. The cDNA sequence encoding for p29 cDNA (GenBank, accession no. AF078931) was used as a query for BLAST search at the *Echinococcus* blast server (available at: http://www.sanger.ac.uk/cgi-bin/blast/submitblast/Echinococcus).

### Accession numbers

The accession number for the known *E. granulosus* s.s. (G1) *p29* cDNA sequence used in this study (Gonzales et al. 2002 [6]) is AF078931 deposited on GenBank. The nucleotide sequences of the *Echinococcus* genus *p29* genes identified in this study have been deposited in GenBank under the following accession numbers: KF528663 (*E. granulosus* s.s. (G1) allele 1), KF528664 (*E. granulosus* s.s. (G1) allele 2), KF528665 (*E. equinus*), KF528666 (*E. ortleppi*), KF528667 (*E. canadensis* (G6)), KF528668 (*E. canadensis* (G7)), KF528669 (*E. canadensis* (G10)), KF528670 (*E. multilocularis*; Swiss isolate), KF528671 (*E. multilocularis*; German isolate), KF528672 (*E. multilocularis*; St. Lawrence Island), KF528673 (*E. multilocularis*; Canada).

## Results

### Molecular characterization of *p29*

**Figure 1:**
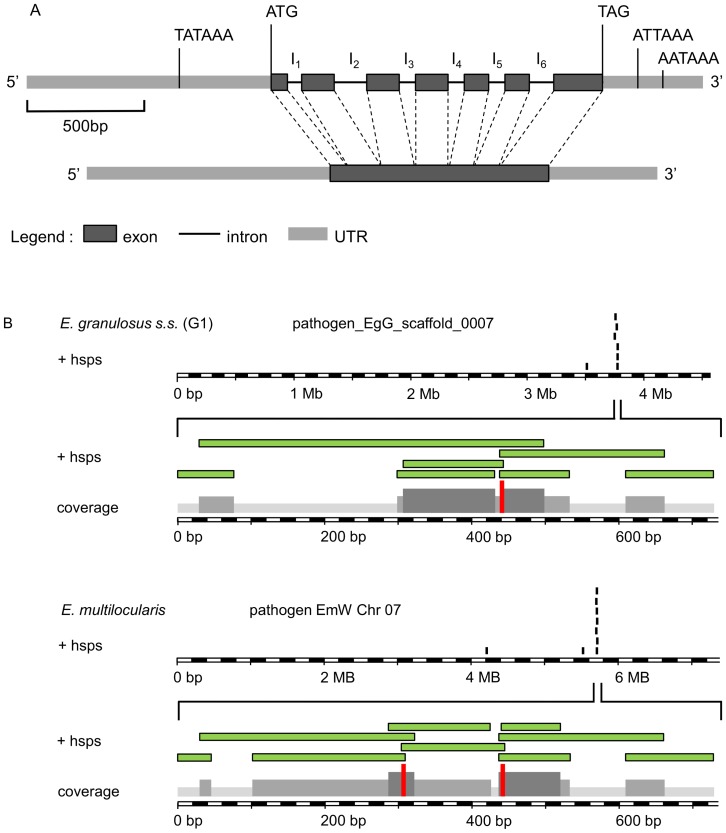
Determination of copy number and molecular structure of the *Echinococcus p29* gene. (**A**) Exon/intron structure analysis of the *Echinococcus p29* gene mapped to the alignments of full-length cDNA with the genomic DNA. The *p29* gene is of 1200 base pairs from the ATG start codon at position +1 to the TAG stop codon at positon +1200 and consists of 7 exons separated by six introns. At position P (−552) upstream of the start codon, we identified a TATA box and a eukaryotic transcriptional regulatory element. Additionally two possible polyadenylation sites were identified downstream of the TAG codon on P (+1365) and P (+1510) positions. (**B**) Graphical output of the BLAST analysis of *p29* cDNA (GenBank, accession no. AF078931) beween *E. granulosus* s.s. (G1) and *E. multilocularis* genomes performed at the *Echinococcus* blast server (available at: (available at: http://www.sanger.ac.uk/cgi-bin/blast/submitblast/Echinococcus). The diagram shows the reads with significant BLAST scores to *p29* cDNA query (+hsps; high score probability). The reads cluster belongs to the same contig; pathogen_EgG_scaffold_0007 and pathogen EmW Chr 07 for *E. granulosus* and *E. multilocualris* respectively. Reads from the same cluster are contiguous and overlapping DNA fragments and when assembled resulted in a single and complete *p29* gene sequence.

The exon/intron organization of *p29* was characterized by comparing (i) the *p29* cDNA sequences isolated from 5 Tunesian *E. granulosus* s.s. (G1) sheep isolates (732 nucleotides, deduced sequence of 238 AA) with (ii) the published genomic sequence. The coding sequence is composed of 1,200 bp, containing seven exons and six introns (Figure 1A). The locations of splice donor/acceptor sites in the introns follow the consensus ‘GT/AG’ rule [36]. Interestingly two different alleles of *p29* were found in the 5 Tunisian sheep-derived *E. granulosus* s.s. (G1) samples. More detailed information on the differences between the alleles A1 and A2 (8 single base mutations) is given below.

The *p29* allele A2 sequence obtained herein is identical to the corresponding sequence of the recently published genome of *E. granulosus* s.s. (G1) [37]. In addition, we identified a putative mRNA initiation site/tataa-box located 552 nucleotides (nt) upstream of the start ATG. According to *E. multilocularis* RNA- Sequencing data [37], the *p29* transcription start point is 70 nt upstream of the ATG initiator codon and in another study [6] 25 nt of 5′ N-terminal region (NTR) has been characterized. Taken together, these information indicate that the transcription of *p29* certainly starts far downstream of the TATA box we found (P -552). Therefore, examination of the regulatory region revealed a double TATA box-like motif (TAAATAA) located 51 and 80 bp upstream of the translated sequence. These two TATA box-like sequences may serve as initiation site for the transcription of *p29*.

Two putative polyadenylation signals ATTAAA and AATAAA located at nt P (+1365) and P (+1510) upstream of the poly (A) tract (Figure 1A).

A BLASTN search with the *E. granulosus p29* DNA sequence on the genomes of *E. granulosus* and *E. multilocularis* revealed that in both species *p29* is present as single copy gene. The *p29 gene* locus of *E. granulosus* is located on the following contig; pathogen_EgG_scaffold_0007 (*E. granulosus* genome assembly database; the systematic gene name for P29 in *E. granulosus* genome is EgrG_000550800) and the *p29 gene* locus of *E. multilocularis* is located on chromosome 07 (EmW Chr 07 from *E. multilocularis* genome assembly, version 4; the systematic gene name for P29 in *E. multilocularis* genome is EmuJ_000550800) of the current assembly versions of respective genome sequences deposited in the Welcome Trust Sanger Institute database (Figure 1B).

To check whether or not *p29* is trans-spliced to the first previously described splicer leader sequence [33], PCR was carried out, and additionally an *E. granulosus* protoscolex specific spliced-leader cDNA library present on the Welcome Trust Sanger Institute database was blasted. PCR was negative and no related sequences were identified in this library.

### P29 protein sequence differences within the *Echinococcus* genus

**Figure 2:**
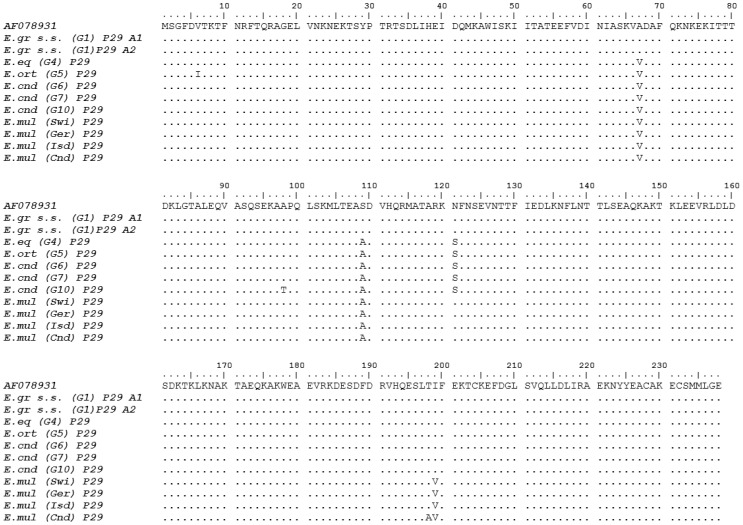
Alignment of amino acid sequences of the P29 *Echinococcus* protein. Two alleles within *E. granulosus* s.s. (G1) for the *p29* gene locus were identified. They code for the same protein and are 100% identical to the published *p29* sequence (GenBank accession no. AF078931). Deduced P29 protein homologs from *E. equinus* (G4), *E. ortleppi* (G5), *E. canadensis* (G6), *E. canadensis* (G7), *E. canadensis* (G10) and *E. multilocularis* (Four isolates from Switzerland, Germany, St. Lawrence Island and Canada) are aligned. The sequence alignment is numbered and the sequences are represented in blocks of 10 AAs. Identical residue sequences are presented in points, and substitutions are presented in letters. Numbers to the left of the sequence corresponds to the AA position at the start of each line.

The P29 amino acid sequences of the *E. granulosus* complex members *E. granulosus* s.s. (G1), *E. equinus* (G4), *E. ortleppi* (G5), *E. canadensis* (G6), *E. canadensis* (G7) and *E. canadensis* (G10) were aligned and a comparative analysis revealed a high (97.9%) conservation of P29 between the species/genotypes (Figure 2).

Three exchanges in the predicted AA sequence discriminate *E. granulosus* s.s. (G1) from the following cluster (*E. equinus* (G4), *E. ortleppi* (G5), *E. canadensis* (G6), *E. canadensis* (G7) and *E. canadensis* (G10)); (i) a substitution of alanine 67 by valine (C-to-T transition at nt 394 in exon 3), (ii) a substitution of serine 109 by alanine (T-to-G transition at nt 592 in exon 4) and (iii) a replacement of asparagine 121 by serine (A-to-G transition at nt 629 in exon 4). Compared to *E. granulosus* s.s. (G1), *E. ortleppi* (G5) showed a single specific substitution of valine 6 by isoleucine (G-to-A transition at nt 16 in exon 1) and *E. canadensis* (G10) showed a single specific substitution of alanine 98 by threonine (G-to-A transition at nt 486 in exon 3).

Compared to *E. granulosus* s.s. (G1), all four *E. multilocularis* isolates presented the two common mutations previously described (Alanine 67 and Serine 109) and one additional substitution of isoleucine 199 by valine (A-to-G transition at nt 997 in exon 6), that specifically differentiates *E. multilocularis* from the whole *E. granulosus* complex. Among most geographically distinct *E. multilocularis* isolates, the P29 AA sequences are highly conserved (99.58%), except the Canadian *E. multilocularis* isolate that includes an additional substitution of threonine 198 by alanine (A to G transition at nt 994 in exon 6).

### P29 Western blot and competition assay

**Figure 3:**
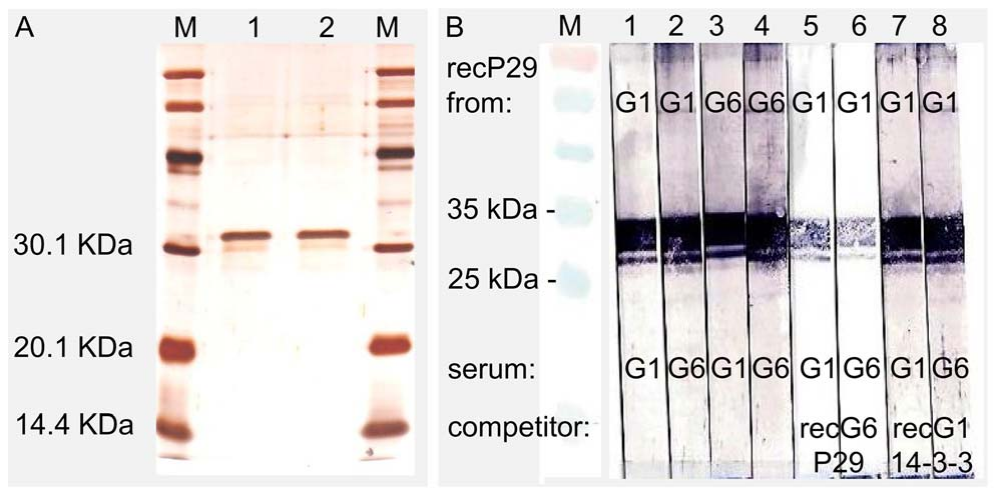
Competitive Western blot analysis with recEg(G1)P29 expressed from *E. granulosus* s.s. (G1) and recEcnd(G6) expressed from *E. canadensis* (G6). (A) Shows a silver stained gel of expressed and purified recombinant antigens; recEg(G1)P29 (lane 1) and recEcnd(G6)P29 (lane 2). (**B**) Recombinant P29 proteins were separated by SDS-PAGE under reducing conditions and blotted onto a nitrocellulose membrane. Human serum from a CE patient either infected with *E. granulosus* s.s. (G1) or *E. canadensis* (G6) were added to recEg(G1)P29 (strip 1 and 2, respectively) or recEg(G6)P29 (strip 3 and 4, respectively). In a competition assay nitrocellulose strips loaded with recEcnd(G1)P29 were incubated with serum from CE patients either infected with *E. granulosus* s.s. (G1) or *E. canadensis* (G6). The sera were pre-incubated with the recombinant expressed competitor recEcnd(G6)P29 (strip 5 and 6) or as a control with recEg14-3-3 (strip 7 and 8). Immune sera were used at dilutions of 1∶100 and competitor/control at concentrations of 1 µg/mL.

RecP29 derived from *E. granulosus* s.s. (G1) (RecEg(G1)P29) and *E. canadensis* (G6) (recEcnd(G6)P29) were expressed and purified. The purity of both recombinant proteins was determined by SDS-PAGE and sub-sequential silver staining to be >90%, as shown in Figure 3A.

Native and recEg(G1)P29 have earlier yielded promising results for post-therapy monitoring of treated CE cases [8]. *E. granulosus* s.s. (G1) followed by *E. canadensis* (G6/G7) are responsible for most human infections worldwide. Thus, a standardized serological test based on recEg(G1)P29, could be useful for all CE patients either infected by *E. granulosus* s.s. (G1) or *E. canadensis* (G6/G7). Here in this study we characterized three main AA substitutions between these two species and we aimed to check whether or not this minor difference has an implication in terms of antigenicity (antibody binding activity), and here especially if sera raised against G6/7 do bind to recEg(G1)P29 and vice-versa.

By Western-blot analysis the serum from a human CE patient infected with *E. canadensis* (G6) as well as 29 out of 34 sera (85%) from humans infected with *E. granulosus* s.s. (G1) reacted with both, recEg(G1)P29 and RecEcnd(G6)P29. However, 5 out of the 34 *E. granulosus* s.s. (G1) sera were negative in both Western-blot assays.

Furthermore, in a competition assay, in Figure 3B, serum from an *E. granulosus* s.s. (G1) patient and serum of another *E. canadensis* (G6) infected patient, were pre- incubated with recEcnd(G6)P29. Subsequently to this prior incubation with recEcnd(G6)P29, these sera are expected to lose their binding capacities in recEgP29 (G1) Western blot assay. As shown in Figure 3B (strip 5 and 6), pre-incubation with recEgP29 (G6) competitor markedly diminished antibody binding activity, but did not completely abrogate anti-P29 reactivity. In fact, it is conceivable that in the competition assay (i) the used concentration of the competitor (here recEgP29 (G6)) was not sufficient to block all anti-Eg(G1)P29 or anti-Ecnd(G6)P29 antibodies in human sera or (ii) the reaction duration (30 min) was too short. In both cases, residual anti-P29 antibodies subsequently reacted with recEg(G1)P29 antigen-coated nitrocellulose strips. To demonstrate that this cross-reactivity of human anti-P29 antibodies with recEg(G1)P29 and recEcnd(G6)P29 is *Echinococcus* P29 antigen-specific, we additionally performed a control competition assay employing out-group recEg14-3-3 from *E. granulosus* s.s. (G1). For the two tested sera, the human anti-P29 antibody reactivity to the recEg(G1)P29 or recEcnd(G6)P29 following a pre-incubation with recEg14-3-3 antigen were not affected, indicating that the binding was indeed specific. Thus, in patients infected with *E. granulosus* s.s. (G1) or with *E. canadensis* (G6), serum anti-P29 antibodies react with both recEg(G1)P29 and recEcnd(G6)P29, demonstrating that minor AA substitutions do not have measurable impact on P29 antigenicity in this test.

### Phylogenetic analysis of *Echinococcus* spp. based on the *p29 gene* locus

**Figure 4:**
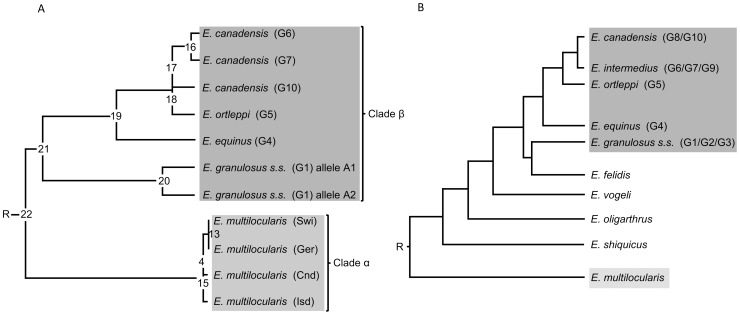
Comparison of phylogenies of *Echinococcus* inferred by maximum likelihood (ML) analysis using gDNA data. (**A**) Maximum likelihood with molecular clock rooted cladogram from this study, based on *p29* gene sequence (exons and introns). (**B**) For comparison a published cladogram based on the DNA sequences of nuclear protein-coding genes is shown [38].

The phylogenetic relationship among the 7 *Echinococcus* taxa *E. granulosus* s.s. (G1), *E. equinus* (G4), *E. ortleppi* (G5), *E. canadensis* (G6), *E. canadensis* (G7), *E. canadensis* (G10) and *E. multilocularis* were assessed mainly by using the multiple nucleotide alignment data representing all of the *p29* genomic sequences. The tree topology inferred from the maximum-likelihood with molecular clock analysis is displayed as a cladogram (Figure 4). The phylogenetic tree identified two major clades (α and β). In the basal position, clade α included all *E. multilocularis* isolates from different geographical areas, whereas clade β consisted of all *E. granulosus* complex members as a monophyletic group. Within clade α, members exhibited a close relationship, particularly between Swiss and German *E. multilocularis* isolates. In contrast, in clade β, at least three divisions became apparent: (**i**) the first separate *E. granulosus* s.s. (G1) (referred as clade β; subgroup 1) from the rest (*E. equinus* (G4), *E. ortleppi* (G5), *E. canadensis* (G6), *E. canadensis* (G7) and *E. canadensis* (G10)) (referred as clade β; subgroup 2). (**ii**) The second separated *E. equinus* (G4) (referred as clade β; subgroup 3) from the clade β; subgroup 4 which clusters *E. ortleppi* (G5), *E. canadensis* (G6), *E. canadensis* (G7) and *E. canadensis* (G10)). (**iii**) The third segregation differentiates *E. ortleppi* (G5) (referred as clade β; subgroup 5) from clade β; subgroup 6 which clusters *E. canadensis* (G6), *E. canadensis* (G7) and *E. canadensis* (G10).

The phylogenetic resolution obtained (Figure 4A) is in agreement with previous studies, which included other nuclear genes such as elongation factor 1 alpha (*ef1*), transforming growth factor beta receptor kinase (*tgf*), thioredoxin peroxidase (*th*), calreticulin (*cal*), and ezrin-radixin-moesin-like protein (*elp*) [38] (Figure 4B).

### Multiplex allele specific polymerase chain reaction (MAS-PCR) for *p29* homo- and heterozygotes genotyping among *E. granulosus* s.s. (G1)

Since we observed two different alleles (A1 and A2) for *p29* in Tunisian *E. granulosus* s.s. (G1) ovine samples, we wanted to assess whether or not one of these two alleles can be associated with a higher infection rate in humans through determining of allele frequency distribution in a in a cohort of patients from Central Tunisia.

We conducted a BLASTN search of these two alleles using blast server for *Echincoccocus* genomes. The BLASTN outputs showed that allele 2 (A2) is identical to the *p29* gene sequence found in the *E. granulosus* s.s. (G1) super contig pathogen_EgG_scaffold_007.

The allele A1 differs from A2 by 8 allelic mutations located on nt positions P157 T/C (silent mutation in exon 2), P355 A/C (intron 2), P372 A/C (intron 2), P505 A/C (intron 3), P542 T/C (intron 3), P559 T/A (intron 3), P730 T/C (intron 4) and P765 C/T (intron 4).

**Figure 5:**
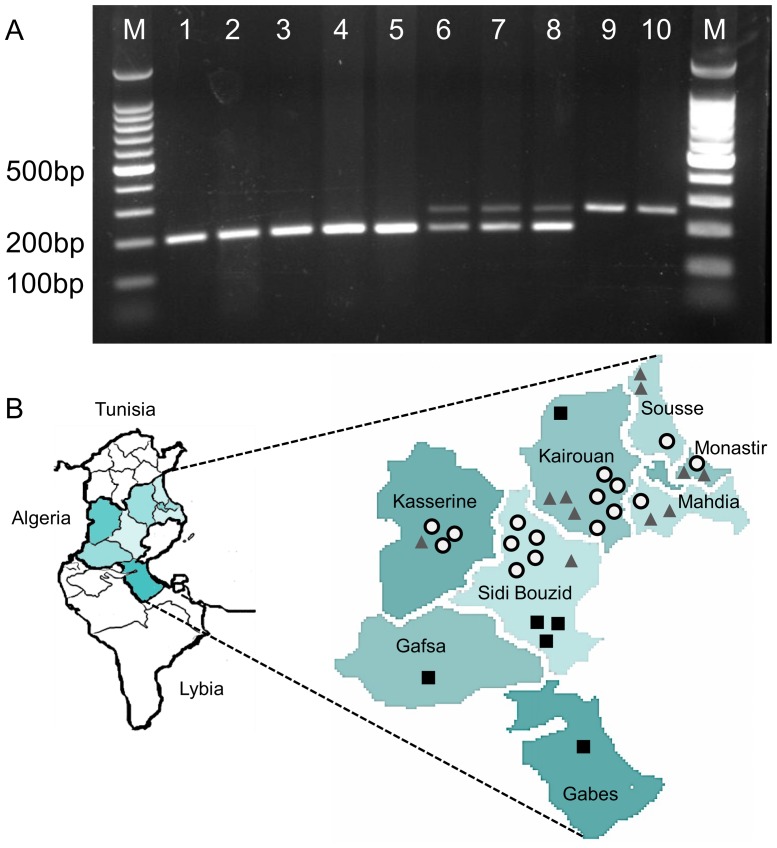
Genetic variability of *p29* within *E. granulosus* s.s. (G1) in Central Tunisia. (**A**) MAS-PCR: *p29* genotype profile of the *E. granulosus* s.s. (G1) by MAS-PCR. Two alleles encoding the P29 protein within *E. granulosus* s.s. (G1) were identified. Result of a MAS-PCR showed homozygotes A1/A1 (lanes 9 and 10), homozygotes A2/A2 (lanes 1–5) and heterozygotes (lanes 6–8), visualized on a 2% agarose gel. M: 100-bp DNA ladder (Promega). (**B**) Location of 34 patients used in this study for the *p29* multiplex allele specific (MAS)-PCR. The main area for human risk is located in Central Tunisia and includes Kairouan, Kasserine and Sidi Bouzid. All isolates were first genotyped as *E. granulosus* s.s. (G1). Samples identified as homozygote A1/A1 are represented by a black square, homozygotes A2/A2 are signified by a grey triangle and heterozygotes are showed by a white circle with black border.

To determine the frequency of homozygotes and heterozygotes in *E. granulosus* s.s. (G1) isolates (see table 3), we created a MAS-PCR. Homozygote A1/A1 and homozygote A2/A2 isolates resulted each in one DNA band in the agarose gel at 195 (figure 5A lane 1–5) and 258 (figure 5 A lane 9 and 10) base pairs respectively. Heterozygous A1/A2 shows two bands corresponding to both alleles (figure 5 lane 6–8). In total, 34 *E. granulosus* s.s. (G1) human isolates were tested. Out of 34 isolates, 16 (47%) were characterized by heterozygote genotype, 6 (18%) by homozygote genotype (A2/A2) and 12 (35%) by homozygote genotype (A1/A1) (see table 3). The location of patients, according to their MAS-PCR profile is shown in Figure 5 B. Frequency of allele A1 (0.6) was higher than the frequency of A2 (0.4).

**Table 3:** Human *E. granulosus* s.s. (G1) allelic frequencies at *p29* loci genotyped with MAS-PCR.

Gouvernorat	Total no. of human cysts	Homozygotes: alleles A1/A1		Homozygotes: alleles A2/A2		Heterozygotes: alleles A1/A2	
	N	N	%	n	%	n	%
Sousse (East-central)	3	0	0	2	67	1	33
Monastir (East-central)	3	0	0	2	67	1	33
Mahdia (East-central)	3	0	0	2	67	1	33
Kairouan (Central)	10	1	10	4	40	5	50
Sidi bouzid (Central)	9	3	33	1	11	5	56
Kasserine (West-central)	4	0	0	1	25	3	75
Gabes (East-sud)	1	1	100	0	0	0	0
Gafsa (West-sud)	1	1	100	0	0	0	0
Total	34	6	18	12	35	16	47

Genotypic frequencies expected under Hardy-Weinberg equilibrium were calculated from allelic frequencies. Deviations observed from expected frequencies were tested by chi-square and showed that the population of 34 *E. granulosus* s.s. (G1) human isolates was in agreement with the Hardy-Weinberg equilibrium (See table 4).

**Table 4:** Observed heterozygosity (H obs) and expected heterozygosity under Hardy-Weinberg equilibrium (H exp) in *p29* locus.

Genotype	H obs	H exp
Homozygote A1/A1	6	5.8
Heterozygote A1/A2	16	16.5
Homozygote A1/A1	12	11.8
Var allele freq:	0.59	
x^2^	0.027755102	
X^2^ test P value	0.867686>0.05	

## Discussion

Immunodiagnostic assays based on full-length or truncated forms (peptides) of recombinant antigens are often hampered by the occurrence of genetic and antigenic variability. The present study was designed to evaluate this possibility for the recombinant *E. granulosus* antigen recEg(G1)P29, which was previously shown to have the potential to be used for the follow-up of young CE patients in view to assess disease evolution and prognosis in post-treatment situations [8]. We investigated whether small sequence changes in recP29 expressed from *E.granulosus* s.s. (G1) have an impact on serological tests used for monitoring of patients infected with other species of the *E. granulosus* complex. As a first step, the *p29* gene locus in different species was characterized with respect to number of gene copies and polymorphism. Results demonstrated that EgP29 in both *E. multilocularis* and *E. granulosus* s.l. is encoded by a single gene, whose transcript does not undergo trans-splicing with the spliced leader reported by Brehm et al. [33]. However, although in this study we did not investigate all 5 SLs that were reported for *E. multilocularis* [37], trans-splicing within *p29* pre-mRNA is completely excluded given that *p29* is not included in the list of *E. multilocularis* trans-spliced genes [37]. This list was established by considering all characterized SLs in *E. multilocularis* [37]. In parasites, antigenic variation often involves specific multigene families devoted to that function (e.g. antigen B multigene family [39]). Here, since we showed that a single gene encodes P29, assumption of antigenic variability in P29 which may due partially to multigene family phenomena, is unlike. In the case of the antigen B multigene family of proteins, the corresponding gene products may represent genetic backgrounds that function for parasite immune evasion mechanisms. Indeed, it was shown that antigen B may plays a role in the escape from early immune response by inhibiting polymorphonuclear cell chemotaxis [40], [41] and by impairing human dendritic cell differentiation. The fact that the P29 is encoded by single-copy gene and is highly conserved within the *Echinococcus* genus (97% identity in AA sequence) minimizes the possibility that this protein plays a role in immune evasion, and it may have another, as yet undefined, role in parasite biology.

An NCBI BLAST search showed that the amino acid sequence of the *E. granulosus* P29 (GenBank, accession no. AAD53328.1 [6]) shares 25% identity with endophilin B1 from many species such as *Bos taurus* (GenBank, accession no. DAA31329.1), *Capitella teleta* (GenBank, accession no. ELT91502.1), *Bubalus bubalis* (GenBank, accession no. XP_006080551.1). In addition, we performed a bioinformatics searches for conserved domains that may be included in the P29 amino acid sequence using the Conserved Domain Database (CDD) of the National Center for Biotechnology Information (NCBI; http://www.ncbi.nlm.nih.gov/Structure/cdd/). Results revealed the presence of the bin1/amphiphysin/Rvs (BAR) domain, which is characteristic of endophilins. Endophilins are major components of clathrin-mediated endocytosis, a crucial cellular mechanism, and they are also involved in other membrane-trafficking events [42]. Thus, P29 possibly carries out an essential function that is highly relevant, and this may explain the high degree of AA sequence conservation among various species of the genus *Echinococcus*.

In this study, we also examined the conservation of the P29 AA sequence among *Echinococcus* spp. The resulting amino acid alignment of *E. granulosus* s.s. (G1), *E. equinus* (G4), *E. ortleppi* (G5), *E. canadensis* (G6), *E. canadensis* (G7), *E. canadensis* (G10) and four isolates of *E. multilocularis* indicated with 97% a high degree of homology. In total, we identified seven polymorphic sites among the 238 AAs of the deduced full length sequence of EgP29.

Western blotting and competition assay analyses suggested that the three different amino acids in the sequence of the P29 proteins from *E. granulosus* s.s. (G1) and *E. canadensis* (G6) have no impact on antigenicity, indicating that they are most likely not located in a major epitopic site and/or antibodies produced against other epitopes are still sufficient to detect both P29 variants in our test system. However, currently there is no information on the structural basis for P29 protein antigenicity and respective epitope mapping. Ben Nouir et al. [8] reported that a minor portion of CE patients remained constantly negative against recEg(G1)P29 from day 0 (before surgery) and until the endpoint of the follow-up period. According to our results, this phenomenon is not related to antigenic variability, but most likely it could be explained by other factors that affect the immune response such as cyst localization. For instance, lung cysts have been shown to not yield an intense humoral immune response [43].

The AA sequence of EgP29(G1) differs from its homologue EcndP29(G6) by 3 AA substitutions, however these minor AA exchanges do not impact its antigenicity, since sera from patients infected with *E. canadensis* (G6) reacted with recEg(G1)P29. The currently most promising vaccine candidate EG95-1(G1) [44] differs also from its homologue in the *E. canadensis* (G6) by few AA variations (5 AA substitutions), but these substitutions result in conformational changes, which lead to a distinct difference in antigenicity between EG95-1G1 and EG95-1G6 [25], [26]. Indeed, antibodies from sheep vaccinated with EG95-1G1 did not react with EG95-1G6 antigen from *E. canadensis* (G6) [26].

Based on comparative sequence analysis of the complete *p29* gene, we investigated the phylogenetic relationships within the *Echinococcus* genus. The phylogenetic resolution obtained in the present analysis is in agreement with previous studies using other nuclear genes [38]. Thus, two major clades are identified, one basal corresponding to *E. multilocularis* isolates and the other one corresponding to all genotypes of *E. granulosus* which were grouped as a monophyletic entity. Therefore, within the *Echinococcus* genus, the *p29* gene exhibits a similar evolutionary dynamic as *ef1*, *tgf*, *th*, *cal* and *elp* genes, a fact that might be due in part to similar selective factors.

Knapp et al. [45] evaluated the potential of single-gene analyses for resolving the taeniid phylogeny by analyzing RNA polymerase II second largest subunit (*rpb2*), phosphoenolpyruvate carboxykinase (*pepck*) and DNA polymerase delta (*pold*) gene markers. Phylogenetic trees based on the analyses of those DNA sequences (*rpb2*, *pepck* and *pold* [45]) yielded low resolutions and were not consistent with the study of Saarma et al. [38] who had been using multiple genes (*ef1*, *tgf*, *th*, *cal* and *elp*). Thus, the authors suggested that the application of a single gene is insufficient to reconstruct phylogeny. However, Knapp et al. analyzed only exons, whereas both, coding and non-coding regions (exons and introns) of P29 (present study) and the 5 nuclear gene markers *ef1*, *tgf*, *th*, *cal* and *elp* [38] were analyzed. This strengthens the usefulness and value of intron datasets, as these provide more information that can be exploited to resolve relationships between the different *Echinococcus* species. Chojnowski et al. [46] showed clearly that introns outperform exons in the analyses of basal avian phylogeny by studying the genes of clathrin heavy chain.

Within *E. granulosus* s.s. (G1), we found two alleles that code for identical P29 proteins. In total, we identified 8 allelic mutations and except the first silenced mutation P157 T/C located on exon 2, all of the seven variations occur within introns number 2, 3 and 4.

Furthermore, we genotyped 34 human isolates of *E. granulosus* s.s. (G1), isolated from 34 young (3–15 years old) patients living in eight different districts of Central Tunisia. By examining the geographical distribution of these two alleles, we observed a difference: allele A1 is more dominant in Central South (Gafsa, Gabes and Sidi Bouzid) and allele A2 is more frequent in Central East (Sousse, Monastir and Mahdia, Figure 5B). However, the number of samples is too limited to draw definitive conclusions, and this explorative observation needs to be confirmed or rejected by including more specimens of defined origin.

A high percentage of heterozygotes (47% (16/34)) comparing to homozygotes (A1/A1 or A2/A2), evokes a possible outbreeding system of *E. granulosus* s.s. (G1) in this part of Central Tunisia, as it was supported by the test of Hardy-Weinberg equilibrium. Asexual reproduction of the *Echinococcus* metacestode stage occurs in intermediate hosts, however in the definitive host, self-fertilization has been proposed to be the predominant reproduction system for adults [47]–[50]. Indeed within the population of *E. granulosus* s.s. (G1), many investigations have not revealed significant heterozygote deficiencies, and this rather supports the cross-fertilization hypothesis [49]. Maillard et al. [51] failed to observe cross-fertilization between the *E. granulosus* (G1) and *E. canadensis* (G6) strains after a mixed experimental infection in dogs. This effect may be due to the fact that *E. granulosus* s.s. (G1) and *E. canadensis* (G6) are two separated species and differences are enough to prevent mating between adult worms, leaving reproduction to self-breeding in this case, and supporting the “different-species” biological theorem.

*E. granulosus* s.s. (G1) is described as the most polymorphic species among the *E. granulosus* (sensu lato) complex, this by using both nuclear and mitochondrial gene analyses [52], [53]. To date, it is still unknown whether genetic variants of *E. granulosus* s.s. (G1) exhibit or not differences in their virulence and infectivity potential to the human host. Even if such segregation is not yet characterized, the populations of homozygote A1/A1 or A2/A2 could differ from each other also in other loci by different allelic combinations, and it can be speculated that both genetic variants show a difference in their virulence. Intra-specific genotyping of human *E. granulosus* s.s. (G1) isolates could contribute to elucidate this open question and the *p29* MAS-PCR assay developed in this study appears as a suitable tool to tackle this question at least in the Central Tunisian area, where the two alleles were identified.

## Conclusions

In summary, we demonstrated a high degree of conservation in the P29 protein/antigen among different species and genotypes of the *Echinococcus* genus. The P29 protein/antigen is encoded by a single gene locus, which exhibits low polymorphism especially in coding regions. Since the recombinant P29 from one species can be recognized by antibodies directed against another species, recombinant P29 such as recEg(G1)P29 can be used as common P29 antigen to detect antibodies derived from P29 of other *Echinococcus* species. Therefore, the recombinant P29 antigen appears useful as a screening tool for post-therapy monitoring in young CE human patients (for older patients, the serological value still needs to be elucidated), independently of the *Echinococcus granulosus* species that was associated with the infection. In this study we tested the two major human infective species *E. granulosus* s.s. (G1) and *E. canadensis* (G6), but due to the very high conservation of P29 between the whole *E. granulosus* complex all other species should also be detectable.
